# “Kankasha” in Kassala: A prospective observational cohort study of the clinical characteristics, epidemiology, genetic origin, and chronic impact of the 2018 epidemic of Chikungunya virus infection in Kassala, Sudan

**DOI:** 10.1371/journal.pntd.0009387

**Published:** 2021-04-30

**Authors:** Hilary Bower, Mubarak el Karsany, Abd Alhadi Adam Hussein Adam, Mubarak Ibrahim Idriss, Ma’aaza Abasher Alzain, Mohamed Elamin Ahmed Alfakiyousif, Rehab Mohamed, Iman Mahmoud, Omer Albadri, Suha Abdulaziz Alnour Mahmoud, Orwa Ibrahim Abdalla, Mawahib Eldigail, Nuha Elagib, Ulrike Arnold, Bernardo Gutierrez, Oliver G. Pybus, Daniel P. Carter, Steven T. Pullan, Shevin T. Jacob, Tajeldin Mohammedein Abdallah, Benedict Gannon, Tom E. Fletcher

**Affiliations:** 1 UK Public Health Rapid Support Team, London School of Hygiene & Tropical Medicine/Public Health England, London, United Kingdom; 2 National Public Health Laboratory, Federal Ministry of Health, Khartoum, Sudan; 3 Karary University, Omdurman, Sudan; 4 University of Kassala, Kassala, Sudan; 5 Laboratory Division, Kassala State Ministry of Health, Kassala, Sudan; 6 Communicable Disease Surveillance & Events Unit, Federal Ministry of Health, Khartoum, Sudan; 7 Health Emergency and Epidemic Control Directorate, Federal Ministry of Health, Khartoum, Sudan; 8 Kassala Teaching Hospital, Kassala, Sudan; 9 Department of Zoology, University of Oxford, Oxford, United Kingdom; 10 National Infection Service, Public Health England, Porton, United Kingdom; 11 Clinical Sciences, Liverpool School of Tropical Medicine, Liverpool, United Kingdom; DoD - AFHSB, UNITED STATES

## Abstract

**Background:**

The public health impact of Chikungunya virus (CHIKV) is often underestimated. Usually considered a mild condition of short duration, recent outbreaks have reported greater incidence of severe illness, fatality, and longer-term disability. In 2018/19, Eastern Sudan experienced the largest epidemic of CHIKV in Africa to date, affecting an estimated 487,600 people. Known locally as Kankasha, this study examines clinical characteristics, risk factors, and phylogenetics of the epidemic in Kassala City.

**Methodology/Principal findings:**

A prospective cohort of 102 adults and 40 children presenting with chikungunya-like illness were enrolled at Kassala Teaching Hospital in October 2018. Clinical information, socio-demographic data, and sera samples were analysed to confirm diagnosis, characterise illness, and identify viral strain. CHIKV infection was confirmed by real-time reverse transcription-PCR in 84.5% (120/142) of participants. Nine (7.5%) CHIKV-positive participants had concurrent Dengue virus (DENV) infection; 34/118 participants (28.8%) had a positive Rapid Diagnostic Test for *Plasmodium falciparum;* six (5.0%) had haemorrhagic symptoms including two children with life-threatening bleeding. One CHIKV-positive participant died with acute renal injury. Age was not associated with severity of illness although CHIKV-infected participants were younger (p = 0.003).

Two to four months post-illness, 63% of adults available for follow-up (30) were still experiencing arthralgia in one or more joints, and 11% remained moderately disabled on Rapid3 assessment. Phylogenetic analysis showed all CHIKV sequences from this study belonged to a single clade within the Indian Ocean Lineage (IOL) of the East/Central/South African (ECSA) genotype. History of contact with an infected person was the only factor associated with infection (p = 0.01), and likely related to being in the same vector environment.

**Conclusions/Significance:**

Vulnerability to CHIKV remains in Kassala and elsewhere in Sudan due to widespread *Aedes aegypti* presence and mosquito-fostering household water storage methods. This study highlights the importance of increasing awareness of the severity and impact of CHIKV outbreaks, and the need for urgent actions to reduce transmission risk in households.

## Author summary

Chikungunya is an arboviral disease transmitted to humans by infected mosquitoes and characterised by fever and arthralgia. Although it is generally considered a short self-limiting infection, long term sequelae and severe disease are increasingly recognised. In 2018/19, Eastern Sudan experienced the largest epidemic of Chikungunya in Africa to date, affecting approximately 500,000 people. We undertook a prospective hospital-based cohort study of patients presenting with undifferentiated febrile illness in Kassala city, Sudan, supported by next-generation sequencing. We confirmed that CHIKV was the dominant pathogen, with positive CHIKV RT-PCR in 85% of patients presenting during the 7-day study period. Dengue virus was also circulating with nine CHIKV RT-PCR-positive patients co-infected, and we identified high rates of *Plasmodium falciparum* infection and CHIKV/*P*.*falciparum* co-infection. Genetic sequencing confirmed Indian Ocean Lineage of the East/Central/South African CHIKV genotype. A quarter of participants available for follow-up (16/60, 26.6%) reported being admitted to hospital including two children with haemorrhage, reflecting the severe phenotype linked to this genotype. Increased understanding of the health and economic burden of Chikungunya is needed, and recognition that severe and occasionally fatal infection exists. With widespread presence of *Ae*. *aegypti* and household water storage practices that encourage mosquito breeding, timely actions will be essential to prevent further large outbreaks.

## Introduction

Chikungunya is often considered a mild illness and its public health impact underestimated in Africa and the Middle East despite the fact that greater severity and long-term sequelae have been reported increasingly in recent years.[1–3]

First isolated in Tanzania in 1952,[4] Chikungunya virus (CHIKV) was implicated in rural outbreaks and sporadic cases across Africa until 1980 when it effectively disappeared. In 2004, however, it reappeared with a vengeance in a pandemic that spread from Kenya to the Indian Ocean islands and Asia, causing millions of infections.[5,6] In central Africa, sizeable CHIKV outbreaks in cities signalled a major shift in transmission to urban settings.[2,3,7–9]

Early symptoms of CHIKV are similar to many tropical febrile illnesses but it is differentiated by debilitating arthralgia, often bilaterally and in multiple joints. Other symptoms include headache, gastrointestinal problems, fatigue, asthenia, peripheral oedema and conjunctivitis.[10] Although most cases improve after 1–2 weeks, recent outbreaks have seen higher incidences of severe illness, including sepsis and cardiac, renal, neurological, skin and ocular manifestations, and of longer-term effects including persistent pain, rheumatic symptoms, depression, and mood and sleep disorders.[11–16] A meta-analysis of chronic symptoms studies in 15 outbreak countries found 43% of cases had not recovered at three months and 21% were unrecovered at 12 months. Long term neurodevelopmental delays have been reported in infants symptomatically infected through vertical transmission.[17,18] Treatment for Chikungunya involves supportive care and anti-inflammatory drugs, but as yet there is no antiviral treatment nor vaccine.[19]

Recent outbreaks have also challenged the view that CHIKV fatality is rare. Case mortality of 10–48% was reported in the Reunion Island outbreak among cohorts of hospitalised patients.[13,16] Central nervous system infections with fatal outcomes have been reported in the Americas.[20] Other locations have documented substantial rises in all-cause deaths during outbreak periods.[20–23]

Hypotheses to explain these changes include persistent immune activation triggered by viral debris, improved surveillance, and genetic mutation, particularly a new variant known as the Indian Ocean Lineage (IOL) which, since the 2004–2006 pandemic, has been linked to greater severity and the highest rates of non-recovery.[24] IOL has also been linked to increased risk of outbreak as some forms contain a mutation that allows CHIKV to use the temperate-dwelling *Aedes albopictus* mosquito as a vector as well the tropical *Aedes aegypti*.[5,24]

In low-resource settings, outbreaks of undifferentiated febrile illness (UFI) are both common and a diagnostic challenge. The Federal Republic of Sudan has seen 12 major outbreaks of UFI since 2012, frequently associated with haemorrhage and high case fatality ratios [25] and a range of high-consequence infectious diseases are endemic or epidemic in Sudan. Crimean-Congo haemorrhagic fever was recently identified as a common pathogen in a retrospective investigation of outbreaks in Darfur.[25] Yellow fever virus epidemics occur [26] and outbreaks of dengue fever are common.[27] The Federal Ministry of Health (FMoH) maintains a sentinel surveillance system and supports individual states with rapid response teams and enhanced diagnostic capability at the National Public Health Laboratory (NPHL) in Khartoum.

**Fig 1:**
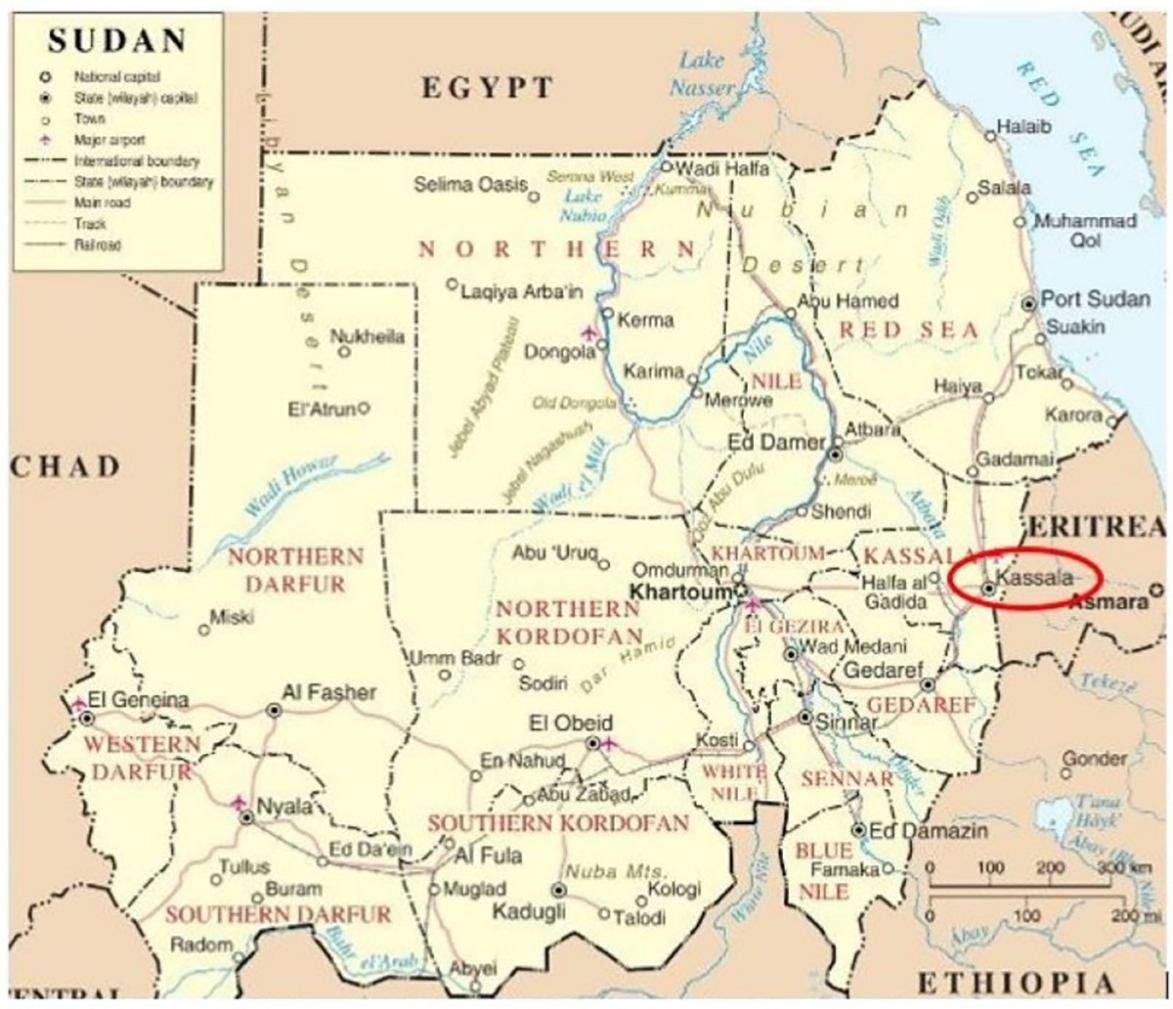
Study location: *Kassala state*, *located in Eastern Sudan*, *600 km the capital city*, *Khartoum*. covers an area of 42,282 km^2^ and has a population 1.8 million inhabitants. Kassala City is the State capital with a population of ~ 400,000. Kassala Teaching Hospital is the State tertiary hospital and provides service for all patients referred from health centres and rural hospitals. The source of the map is: https://commons.wikimedia.org/w/index.php?curid=16093732.

In late July 2018, the sentinel surveillance in Kassala State (Fig 1) detected an increased frequency of UFI, and CHIKV was identified by the NPHL in the blood sample of a traveller to Kassala city from nearby Red Sea State. In mid-September reports of cases with more severe symptoms, including haemorrhage, raised fears that another pathogen might be involved. Using a pre-prepared, locally and internationally-approved protocol, FMoH and Kassala Teaching Hospital (KTH), supported by Kassala University and the UK Public Health Rapid Support Team, deployed a small pre-trained study team of clinical, epidemiology and laboratory staff to investigate the outbreak syndrome, confirm the outbreak pathogen, and sequence the outbreak strain.

## Methods

### Ethics statement

Ethical approval was granted by the FMoH Technical Review Board, and Ethics Committees of Karary University, Khartoum (03 August 2017) and the London School of Hygiene & Tropical Medicine (Ref: 11930). All participants or parents/guardians of children aged under 18 years provided written informed consent and assent was sought from children aged 12–18.

### Participant recruitment

The study was a prospective hospital-based observational cohort of consecutive patients presenting to the Kassala Teaching Hospital medical and paediatrics outpatient departments between 10th and 16th October 2018. Recruitment criteria were individuals of any age or sex with symptoms consistent with the KTH case definition for Chikungunya, namely history of fever or a measured fever of >37.5°C (tympanic), and at least three of the following clinical features: headache, anorexia, lethargy, aching muscles or joints, breathing difficulties, vomiting, diarrhoea, stomach pain, difficulty swallowing, and hiccups, or bleeding of any kind. Patients with a positive malaria RDT were enrolled if they fit the study syndrome in case of co-infection. Participants were enrolled at presentation and blood samples taken immediately. Epidemiological information, clinical symptoms at presentation and subsequent laboratory results were recorded on standardised pro-formas. The follow-up component took place 70–120 days after enrolment (Jan-Feb 2019) depending on the availability of participants. Adults who responded to phone follow-up provided convalescent samples and completed the WHO-validated Routine Assessment of Patient Index Data 3 (RAPID3) disability and pain survey.[28] Children were not asked to return due to common reluctance to allow blood-draw from healthy children, but outcome and duration of hospital stay were confirmed with parents by phone. All contactable participants were given their RT-PCR test results.

The study protocol (available at https://doi.org/10.17037/DATA.00002018) was based on the International Severe Acute Respiratory and Emerging Infections Consortium (ISARIC) Clinical Characterization Protocol for Severe Emerging Infections and adapted to context.[29] The sample size of 140 cases was informed by literature^3^ and feasible recruitment in the urgent time frame (7 days).

### Laboratory analysis

Haematology (Mindaray 3000 Plus, China), biochemistry analysis (Biosystem BTS 310, Germany), and malaria rapid diagnostic tests (*P*. *falciparum* RDT, SD Bioline, USA) were performed on all participants. Plasma samples were frozen at -80°C and transported under liquid nitrogen to the NPHL in Khartoum where nucleic acid was extracted (QIAamp Viral RNA Mini kit, Qiagen, Germany) and RT-PCR performed to detect CHIKV RNA (RealStar Chikungunya RT-PCR Kit 2.0, Altona Diagnostics, Germany) and DENV RNA (RealStar Dengue RT-PCR Kit 2.0, Altona Diagnostics, Germany) using Rotor Gene Q or Corbett RG6000 thermocyclers. Exposure to CHIKV and DENV was assayed by anti-chikungunya and anti-dengue IgM and IgG indirect ELISA (Euroimmun, Germany).

Due to limited availability of anti-chikungunya IgM ELISA kits, CHIKV RT-PCR-negative samples were prioritised for IgM testing, followed by 69 CHIKV RT-PCR-positives in order of recruitment. All samples were tested for CHIKV IgG. An Ebolavirus RT-PCR (RealStar Ebolavirus RT-PCR Kit 1.0, Altona Diagnostics, Germany) was performed on seven samples from patients with haemorrhagic symptoms, collected prior to the study to rule out this pathogen: all were negative.

### Data management

All patient data were entered into encrypted software and analysed with STATA (StataCorp LLC, USA, V14.2). Descriptive statistics are expressed as medians and Inter-Quartile Range (IQR) or means and standard deviations (SD) for continuous variables, and frequencies and proportions for categorical variables. Fisher’s exact, Chi^2^, T-tests and Spearman’s Rank Order coefficients were used to assess association and correlation related to CHIKV RT-PCR result. Significance was set at p<0.05. Only RT-PCR results were used in comparative analyses.

### Genetic sequencing

Complementary DNA (cDNA) was prepared and Sequence Independent Single Primer Amplification (SISPA) performed prior to library preparation using Oxford Nanopore kits (SQK-LSK108). Sequencing on flow cells (FLO-MIN106) was done using the MinION (Oxford Nanopore Technologies) as previously described.[30] Thirty samples were sequenced at NPHL and analysed using Albacore 1.2 to basecall, Mash Screen [31] was used to identify the most closely matched genome on Genbank, and a reference guided genome assembly constructed as described previously.[30] The first seven sequences were used to construct a basic phylogenetic analysis using MEGA version 6 in order to identify the Kassala strain in Sudan. With FMoH permission, sample aliquots were also transferred to PHE Porton and sequenced using the same methods. The resulting viral genetic sequences were sent to Oxford University Department of Zoology for phylogenetic analysis.

Whole genome sequences were aligned with publicly available complete CHIKV genomes belonging to the ECSA genotype using MAFFT *v7*.*450* implemented in Geneious R8.[32] A maximum likelihood (ML) phylogenetic tree was estimated using RAxML 8.0 [33] under a GTR substitution model with a Gamma model of among-site rate heterogeneity. The estimated ML phylogeny was midpoint rooted, and node support was assessed through 100 ML bootstrap replicates. The geographic location and amino acid identity at site E1-226 of each sequence were annotated in the tree to explore the phylogenetic distribution of those traits.

## Results

**Fig 2:**
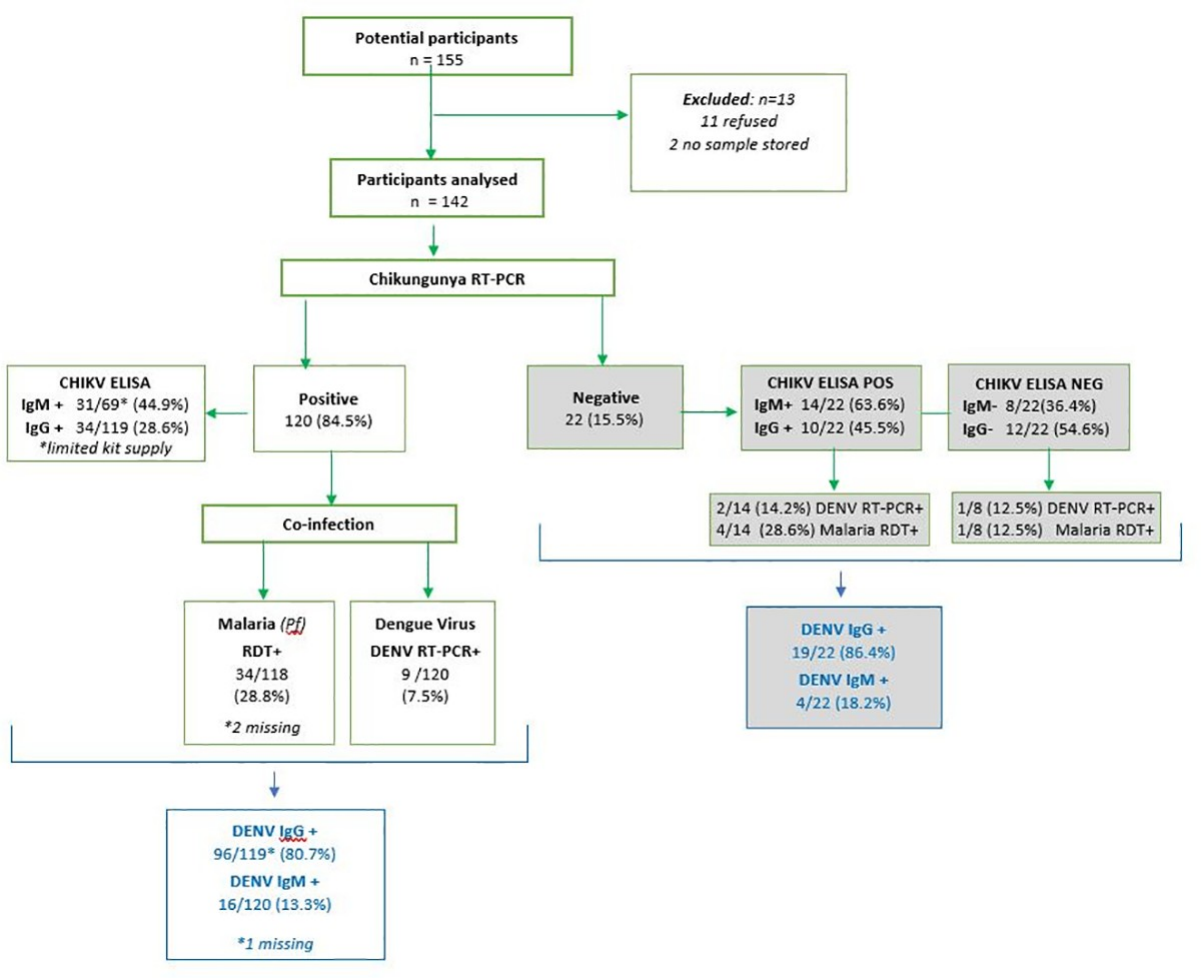
Flow diagram of study participants and virological findings: *CHIKV*: *Chikungunya; DENV*: *Dengue Virus*. ELISA IgM testing was limited by kit availability: CHIKV RT-PCR-negative samples were prioritised, followed 69 CHIKV RT-PCR positive samples in order of recruitment.

Of 155 patients approached over seven days, 102 adults and 40 children were included in the analysis. Eleven patients or their guardians refused to participate, and two cases whose samples were lost were excluded. (Fig 2).

Participants were aged 4 months to 70 years (mean 27 years, SD 17.6); 47.9% were female, none were pregnant (Table 1). Almost half (46.1%, 65/141) were people likely to spend more time in a household compound (e.g. housewives, unemployed, retired, children under 5). Median household size was 8. Two-thirds of participants lived in brick/concrete houses with their own well and sanitation, the remainder lived in less permanent material structures with shared water and sanitation. A third kept animals in their compound.

**Table 1:** Participant characteristics by Chikungunya (CHIKV) RT-PCR result.

	total	CHIKV positive (n = 120)	*%*	CHIKV negative (n = 22)	*%*
Sex					
Female	47.9%	56	*82*.*3*	12	*17*.*7*
Male	52.1%	64	*86*.*5*	10	*13*.*5*
Age (y), median [IQR]		22	*14–40*	37	*21–54*
Age Group (y) (n = 141)					
<2	7	7	*5*.*9*	0	*0*.*0*
2–4	6	5	*4*.*2*	1	*4*.*5*
5–14	24	22	*18*.*5*	2	*9*.*1*
15–29	51	46	*38*.*7*	5	*22*.*7*
30–49	33	26	*21*.*8*	7	*31*.*8*
50+	20	13	*10*.*9*	7	*31*.*8*
Occupation (n = 141)					
< 5 child	13	12	*10*.*0*	1	*4*.*8*
Schoolchild/student	49	43	*35*.*8*	6	*28*.*6*
Housewife	31	26	*21*.*7*	5	*23*.*8*
Farmer/Outdoor worker	15	12	*10*.*0*	3	*14*.*3*
Professional/Business	7	7	*5*.*8*	0	*0*.*0*
Health worker	5	3	*2*.*5*	2	*9*.*5*
Retired/unemployed	21	17	*14*.*2*	4	*19*.*0*
Median household size (IQR) n = 102	8	8	*6–11*	7	*5–9*

Notes: Missing data: age 1, occupation 1, # in household 40. IQR: Interquartile Range

Most participants who provided residence location (69%, 80/116) came from Kassala City sectors 2, 3, 4 and 5 on the banks of the seasonal River Gash, coinciding with areas of greatest flooding in the 2018 rainy season, and with highest case reports during the epidemic. A further 26% (30/116) were from rural areas up to 1.5 hours’ drive away.

### Laboratory results

Of 142 participants, 120 were confirmed positive for CHIKV infection by RT-PCR, giving the case definition used a positive predictive value of 84.5%. CHIKV RT-PCR cycle threshold (Ct) values ranged from 12–37 (mean 27.3, SD 7.6); over a quarter (27.5%) had a Ct value corresponding with a high viral load (Ct<20) (Table 2). A significant positive correlation existed between Ct value and both the number of days between onset and recruitment/sampling (Spearman r = 0.37, p<0.001) and lymphocyte count (r = 0.23, p = 0.012). No other significant correlations were found. Prolonged viremia was observed in seven CHIKV RT-PCR-positives who presented 10 days after disease onset. Of 91 participants tested for CHIKV IgM antibodies, 45 were positive including 14 participants who were negative on CHIKV RT-PCR (Fig 2). There are several possible explanations for the IgM positive but RT-PCR negative cases: an acute or recent infection outside the viraemic phase of infection and therefore outside the diagnostic window for RT-PCR, prior CHIKV infection but unrelated to this outbreak (as IgM may persist for months [34]) or a false assay result.

**Table 2:** Clinical symptoms and cycle threshold (Ct) values of participants at presentation by CHIKV RT-PCR result (n = 142).

	CHIKV positive		CHIKV negative	
**Days from onset to presentation (median)**	2	*IQR 1–4*	2	*IQR 1–5*
**Symptoms at presentation**		*%*		*%*
Fever/history of fever	117	*97*.*5*	22	*100*.*0*
Any bleeding	6	*5*.*0*	1	*4*.*8*
Headache	97	*80*.*8*	17	*85*.*0*
Joint pain	93	*77*.*5*	15	*75*.*0*
Fatigue	85	*70*.*8*	17	*81*.*0*
Muscle pain	66	*55*.*0*	16	*76*.*2*
Back pain	55	*45*.*8*	9	*42*.*9*
Loss of appetite	46	*38*.*3*	6	*28*.*6*
Vomiting	45	*37*.*5*	8	*38*.*1*
Abdominal pain	32	*26*.*7*	8	*38*.*1*
Lack of strength	25	*20*.*8*	4	*100*.*0*
Dizziness	18	*15*.*0*	3	*14*.*3*
Chest pain	17	*14*.*2*	3	*15*.*0*
Cough	17	*14*.*2*	2	*9*.*5*
Rash	13	*10*.*8*	2	*9*.*5*
Diarrhoea	11	*9*.*2*	2	*10*.*0*
Dysphagia	9	*7*.*5*	3	*15*.*0*
Dehydration	9	*7*.*5*	1	*33*.*3*
Shortness of breath	7	*5*.*8*	2	*10*.*0*
Conjunctivitis	7	*5*.*8*	3	*15*.*0*
Sore throat	6	*5*.*0*	0	*0*.*0*
Confusion	3	*2*.*5*	0	*0*.*0*
**RT-PCR cycle threshold (Ct)**				
High (Ct <20)	33	*27*.*5*	-	
Moderate (Ct 20–29)	24	*20*.*0*	-	
Low (Ct 30–38)	63	*52*.*5*	-	
**Other pathology**				
Malaria RDT positive	34/118	*28*.*8*	5/22	*22*.*7*
DENV positive (RT-PCR)	9/120	*7*.*5*	3/22	*13*.*6*

Notes: RDT Rapid Diagnostic Test. Missing data: fever 3; onset date 3; malaria RDT 2.

Nine CHIKV RT-PCR-positive participants (7.5%) were co-infected with DENV, defined as being positive on DENV RT-PCR. A further 14 CHIKV RT-PCR-positive participants had evidence of DENV IgM antibodies, potentially indicating current or recent DENV infection, or due to cross reactivity of other flavivirus infections. Of all samples (CHIKV-positive and negative), 81.6% (115/141) were DENV IgG-positive suggesting previous infection with DENV or cross-reactive flaviviruses. Of the 118 CHIKV RT-PCR-positive participants with a *falciparum* malaria RDT result, 28.8% were positive, with the highest proportion in those aged 15–29 years. No participants were co-infected with all three pathogens.

Among the 69 CHIKV RT-PCR-positive participants tested for CHIKV IgM, 31 (44.9%) were positive. Of the 119 CHIKV RT-PCR tested for CHIKV IgG, 34 (28.6%) were positive (Fig 2). Of the 22 CHIKV RT-PCR-negative participants, 14 (63.6%) were CHIKV-IgM positive and 10 (45.5%) were CHIKV IgG positive. Participants who were CHIKV-positive on both RT-PCR and IgM ELISA presented later in illness course than those who were CHIKV RT-PCR-positive and IgM-negative (p<0.001). This finding was replicated with CHIKV RT-PCR & IgG-positive patients who presented later than CHIKV IgG-negative patients (p = 0.002).

### Clinical presentation

Median delay from symptom onset to presentation was 2 days (IQR 1–4, n = 139) with no difference by CHIKV diagnosis or age (Table 2).

Most common symptoms at presentation among CHIKV RT-PCR-positive participants were current or history of fever (97.5%, 2.5% data unrecorded), headache (88.2%), fatigue (82.5%), muscle, joint and back pain (66.0%, 83.8%, 50.9% respectively), loss of appetite (40.4%) and vomiting (40.2%). Patients who were found to be CHIKV RT-PCR-negative had similar presenting symptoms. Five percent (6/120) of CHIKV RT-PCR-positive participants reported bleeding, including haematemesis (4), oral bleeding (2), epistaxis (3), petechiae (1), haemoptysis (1) and melaena (1). None of those with haemorrhagic symptoms were co-infected with DENV but one–a 9-year-old child with haematemesis–was co-infected with malaria. Eighteen of 78 CHIKV RT-PCR-positive adults (23.1%) were hypotensive (systolic blood pressure <100 mmHg) and seven (9.0%) were tachycardic (pulse >100), one of whom was co-infected with DENV. Only one person was both tachycardic and hypotensive.

Information on duration of admission was collected at follow-up. Of the 30 child guardians and 30 adult participants who responded to follow-up, 16 (26.6%) reported being admitted. All were CHIKV RT-PCR positive and none were coinfected with DENV, but 60% (7/12 children, 2/4 adults) had a positive *falciparum* malaria RDT. Children were more likely to be admitted than adults (40.0% vs 13.3%, Fisher’s p = 0.04) and for longer: mean 4.7 days (sd 4.1) compared to 2.3 days (sd 1.3) for adults. Both adults and children were more likely to be admitted if they were malaria positive (p = 0.002), and children if they were bleeding (p = 0.054). One of the four children admitted for seven or more days was malaria positive.

In analysis of blood chemistry, a significant difference was found in mean haematocrit levels between CHIKV RT-PCR-positive (mean 35.9, SD = 6.27) and negative participants (mean 38.9, SD = 7.41, p = 0.05). There were no other significant differences in blood chemistry. Heart rate and blood pressure were similar in both groups. In the CHIKV RT-PCR-positive cohort median haematology and biochemistry results were all within normal ranges (Table 3). Leucopenia (white blood count < 4 x 10^9^/L) was observed in 29% (35/120) and lymphopenia (< 1 x 10^9^/L) observed in 50.7% (61/120). Elevated AST (>40IU/L) was observed in 22.5% (27/120) with six participants at levels> 100 IU/L (max 259 IU/L). ALT levels >51 IU/L were seen in seven participants with four recording levels >100 (max 150 IU/l). Acute kidney injury was observed in one fatal case. Platelet counts < 100 x 10^9^/L were recorded in 23/120 CHIKV RT-PCR-positive participants, of whom 14/23 (60.9%) were also *falciparum* malaria RDT-positive and none were DENV PCR-positive. Platelet counts < 50 x 10^9^/L were observed in six participants, three of whom also had positive malaria RDT.

**Table 3:** Mean clinical parameters of participants at presentation by CHIKV RT-PCR result.

	CHIKV positive	SD	*n*	CHIKV negative	SD	*n*
Heart rate (beats/min)	**91.2**	19.2	*114*	**83.5**	15.8	*21*
Respiratory rate	**20.4**	2.9	*12*	**18.0**	n/a	*1*
Systolic pressure	**102.9**	20.2	*78*	**106.3**	12.5	*20*
Diastolic pressure	**68.0**	13.1	*78*	**70.2**	7.5	*20*
Haemoglobin (g/dL)	**11.6**	2.2	*120*	**12.5**	2.5	*22*
Haematocrit (%)*	**35.9**	6.3	*119*	**38.9**	7.4	*22*
WBC (10^3^/uL)	**6.1**	3.1	*120*	**4.8**	2.2	*22*
Lymphocytes (10^3^/uL)	**2.1**	2.4	*118*	**2.9**	6.6	*22*
Platelets (10^9^/L)	**227.0**	143.3	*120*	**190.6**	131.0	*22*
Nitrogen oxide	**3.8**	2.0	*113*	**2.9**	1.7	*22*
Urea (mg/dL)	**24.8**	14.9	*119*	**26.1**	11.3	*22*
Creatinine (mg/dL)	**0.8**	0.7	*119*	**0.9**	0.2	*22*
Bilirubin	**0.7**	1.5	*119*	**0.9**	1.3	*22*
AST (IU/L)	**36.0**	33.9	*119*	**28.2**	17.4	*22*
ALT (IU/L)	**23.1**	23.4	*118*	**21.5**	14.4	*22*
Albumin	**14.9**	6.8	*69*	**17.6**	6.4	*17*

Notes: WBC White Blood Cells; AST Aspartate aminotransferase; ALT Alanine aminotransferase.

*Significant difference p = 0.049

Compared to those with a single infection, CHIKV/DENV co-infected participants had higher mean systolic blood pressure (p = 0.03) haemoglobin (p = 0.01), haematocrit (p = 0.03), bilirubin (p = 0.002) and albumin (p = <0.001). They were also more likely to present with back pain than those with CHIKV alone (p = 0.003).

### Severe or fatal disease

The study recruited one case with a fatal outcome–a male teacher aged 52 transferred to KTH 13 days after onset of symptoms. He was CHIKV RT-PCR-positive with a Ct of 30 and no coinfection. At admission he had petechiae and minor bruising but no other evidence of bleeding. He died from sepsis complicated by disseminated intravascular coagulation and acute kidney injury (Blood/Urea/Nitrogen (BUN) 154 mg/dL, creatinine 7.8 mg/dL, AST 259 IU/L, ALT 59 IU/L, platelets 111 x 10^9^/L).

Two children *in extremis* with significant haemorrhage were recruited at the paediatric department. Both were CHIKV RT-PCR-positive without coinfection. The 16-year-old was admitted on day 2 of illness with a history of fatigue, headache, back pain, anorexia, oral mucosal haemorrhage, and profuse epistaxis. He was in shock (pulse 120, BP 84/50, respiratory rate 25) with a platelet count of 10 x 10^9^/L, Hb 12.5 g/dL, WBC 10.5 x 10^3^/uL, HCT 38.9%, AST 47 IU/L, ALT 24 IU/L, BUN 26 mg/dL, creatinine 0.8 mg/dL and CHIKV RT-PCR Ct 29.63. He was resuscitated with blood and platelet transfusion, underwent nasal packing, and was discharged after 15 days. The second child aged 7 years presented on day 0 of illness with epistaxis, haematemesis, melaena, bruising and anorexia. Her pulse was 130 and Hb 10.2 g/dL, WBC 4.6 x 10^3^/uL, platelet count 20 x 10^9^/L, HCT 30.7%, urea 22 mg/dL, creatinine 0.5 mg/dL, AST 175 IU/L, ALT 129 IU/L and CHIKV RT-PCR Ct of 26.8. She was also resuscitated with blood and platelets and discharged well after nine days’ admission.

### Factors associated with CHIKV positivity

The mean age of CHIKV RT-PCR-positive patients was significantly less than CHIKV RT-PCR-negative patients (p = 0.003). CHIKV RT-PCR-positives were also more likely to report having been in contact with someone who was ill (p = 0.01) but as CHIKV is not transmitted person-to-person, this finding likely relates to being in the same vector environment. No associations were found with any other exposure including mass gatherings, funerals/corpses, or contact with livestock, birds or rodents.

### Convalescent follow-up

Thirty (29.7%) of the 102 adult participants were available for follow-up, 29 (28.7%) refused, and 42 (41.6%) could not be reached. Median time to follow-up for adults was 75 days (IQR 71–94) after enrolment; 52% were male. Parents of 30 of the 40 child participants responded to contact. All 60 participants followed-up were CHIKV RT-PCR-positive at baseline. Of adults followed-up 43.3% (13/30) had spent 1–2 weeks off work and 20.0% (6/30) 2 or more weeks (Table 4). Twenty-three of the 30 adult convalescent samples collected (76.7%) were IgG positive: 10 were IgG-positive in both acute and convalescent samples, 13 had seroconverted since recruitment, and seven remained IgG negative. Children were not sampled.

**Table 4:** Impact of CHIKV infection on work and disability 70–120 days after acute infection.

	Adults (n = 101)	*%/IQR*	Children (n = 40)	*%/IQR*	p (Fisher)
**Follow-up response rate**	30	*29*.*7%*	30	*75*.*0%*	
**Median days to follow-up**	75	*IQR 71–94*	-	*-*	
**Hospital care (n = 30)**					
Discharged the same day	26	*86*.*7%*	18	*60*.*0%*	p = 0.039
Admitted for at least 1 day	4	*13*.*3%*	12	*40*.*0%*	
Median days admitted	2	*IQR 1*.*5–3*	3	*IQR 2–7*	
**Absence from work**					
< 1 week	11	*36*.*7%*			
1–2 weeks	13	*43*.*3%*			
3–4 weeks	4	*13*.*3%*			
>4 weeks	2	*6*.*7%*			
**RAPID3 disability score (n = 28)**					
Near remission	8	*28*.*6%*			
Low disability	17	*60*.*7%*			
Moderate disability	3	*10*.*7%*			
**Current presence of pain (n = 30)**	19	*63*.*3%*			
**Location of pain** *(multiple possible***)**					
Knee	14	*46*.*7%*			
Ankle	11	*36*.*7%*			
Wrist	9	*30*.*0%*			
Shoulder	8	*26*.*7%*			
Fingers	7	*23*.*3%*			
**Median pain perception score**	4	*IQR 2–5*			
**Median wellbeing score**	7	*IQR 3–8*			

Notes: Pain perception and wellbeing are scored on a 0–10 scale with 10 being the worst pain and feeling the worst. IQR: Inter Quartile Range

### Chronic disability

Only adult follow-up participants were surveyed for ongoing disability; median age was 27 years (IQR 24–45). Of these, 63.3% (19/30) were still experiencing arthralgia, most frequently in the knee (46.7% 14/30), ankle (36.7% 11/30) wrist (30.0% 9/30) and shoulder (26.7% 8/30). Asked to rank pain and general wellbeing over the previous week on a scale where 10 was the most pain/most unwell, respondents reported a median pain score of 4 (IQR 2–5) and median wellbeing score of 7 (IQR 3–8) (Table 4). A third (36.6%, 11/30) indicated their pain was greater or the same as during the acute phase of their illness, and even those with less pain than before reported continuing to take painkillers, anti-inflammatories, corticosteroids, or all three, for their condition. In total, 60% continued to take medication.

Of the 28 respondents who completed the RAPID3 disability survey, three-quarters reported a continuing effect on day-to-day activities, including difficulties getting out of bed (28%), walking 2 miles (35%), and getting in and out of a vehicle (17%), while 10–15% reported difficulty with activities such as turning on taps, bending and sleeping well. Rapid3 scaling indicated that, 70–120 days after their acute illness, 10.7% (3/28) of respondents were experiencing moderately severe disability and 60.7% (17/28) low severity disability. The remaining eight (28.6%) respondents were considered nearly recovered (Table 4). Neither age, sex, level of viremia on admission, or pre-existing conditions such as diabetes, hypertension and osteoarthritis, were associated with level of residual disability, or duration of absence from work.

### Genetic analysis

**Fig 3:**
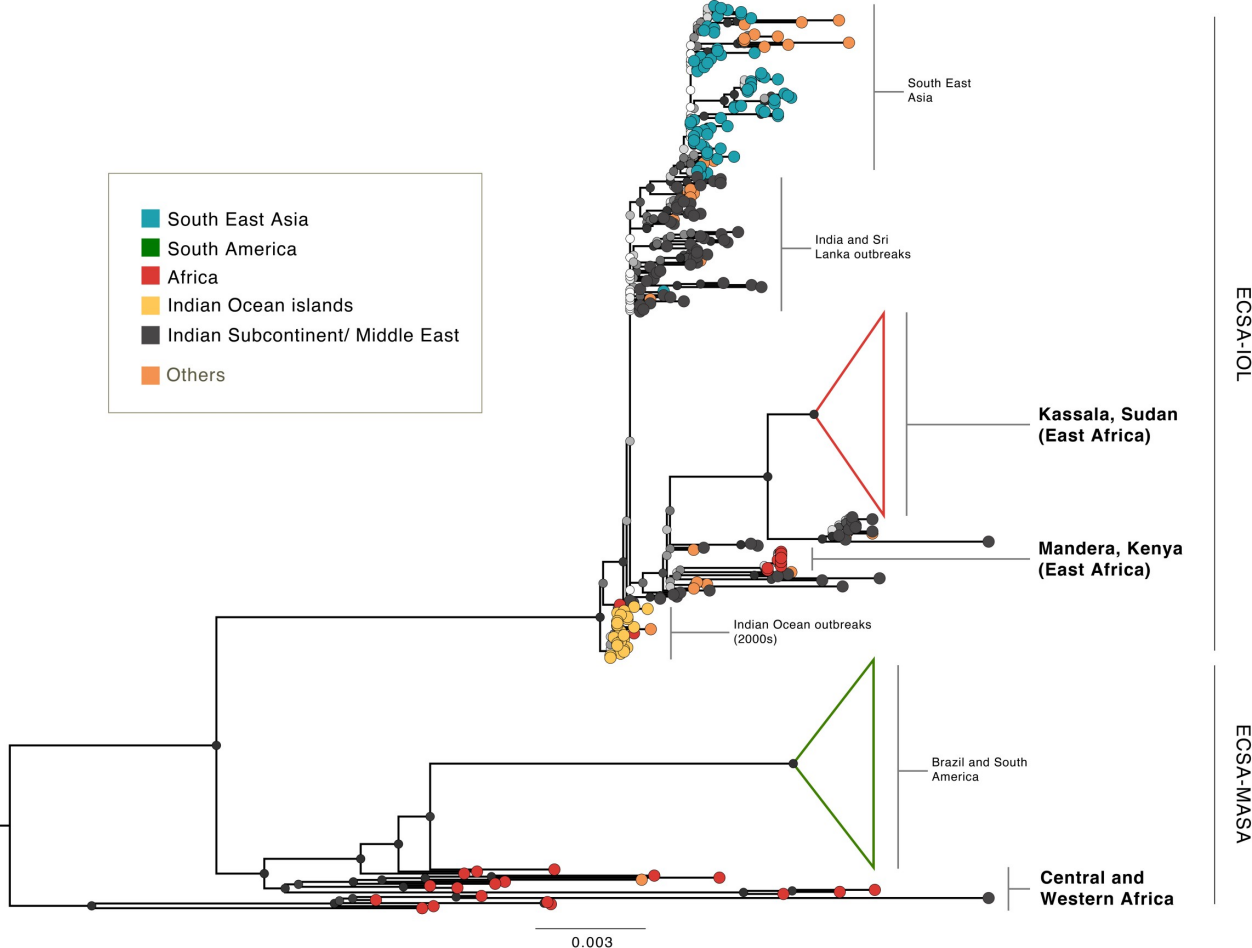
Maximum likelihood phylogenetic tree of the ECSA genotype and its two distinct lineages showing the location of the Kassala 2018/19 strain: *Maximum likelihood phylogenetic tree of the East/Central/South African (ECSA) genotype and its two distinct lineages*: *the Indian Ocean Lineage (IOL) and the Middle African/ South American (MASA) lineage*. The monophyletic Kassala epidemic clade groups with sequences that originate from the Middle East and India and represents a distinct lineage from other African CHIKV variants. Node support values were evaluated using 100 bootstrap replicates and are illustrated using colour at nodes (with white representing 0 and black representing 100). The 2016 Mandera outbreak also represents a possible introduction from the Indian subcontinent and appears to be an independent event from the Kassala epidemic. Neither the Mandera or Kassala outbreak contained the A226V albopictus adaptive variant.

Virus genome sequencing found that all CHIKV RT-PCR-positive samples belonged to a single monophyletic cluster in the Indian Ocean Lineage (IOL) of the ECSA genotype of CHIKV. (Fig 3) Between 84–95% (mean 93%) genome coverage was achieved at least x20 depth across all samples (Genbank Accession Nos. MW161364-161460). This clade is distinct from those previously identified in Western and Central Africa, suggesting that the Kassala outbreak was caused by an independent introduction of an IOL strain into the region. This might have occurred via the Middle East or Indian Subcontinent since the Kassala sequences are closely related (i) to the Henan and Shivane variants identified in China and Hong Kong, which originated in returning travellers from India/Pakistan, and (ii) to a clade that includes sequences from a 2016 outbreak in Pakistan and other sequences from India and Bangladesh. Absence of the A226V mutation—a mutation associated with the capacity of the virus to infect *Ae*. *Albopictus* [35,36]—confirms *Ae*. *aegypti* as the most likely outbreak vector species.(S1 Text)

## Discussion

Our phylogenetic analysis suggests the unprecedented Chikungunya epidemic that took place in the Eastern states of Sudan in 2018–19 was caused by an independent introduction of a CHIKV IOL virus, of the same lineage responsible for the outbreaks observed worldwide during 2004–2007 and linked to more severe manifestations of CHIKV disease.[24]

Over the nine months of the epidemic, Kassala State and Red Sea State reported 48,763 cases of chikungunya. Seroprevalence surveys in other outbreak locations, however, suggest only 2–15% of cases are captured by surveillance and reporting systems.[37–40] A conservative estimate of 10% of cases captured suggests some 487,600 cases occurred in the Kassala and Red Sea State epidemic, making it the largest outbreak of Chikungunya in Africa and the Middle East to date. There are multiple reasons to suspect substantial under-reporting and under-diagnosis in this outbreak including: use of a sentinel surveillance system normally intended to raise an alert rather than capture every case (one study reported a ratio of 67 symptomatic cases for every one picked up by the sentinel system [40]), population reluctance to seek medical attention at hospitals due to cost and limited treatment options, limited diagnostic resources, absence of data collection in private clinics, and the range of CHIKV illness severity.

Made possible by the established research collaboration between the FMoH and the UK Public Health Rapid Support Team on outbreak response, this study utilised pre-approved protocols, pre-trained staff and, for the first time within Sudan, next generation sequencing technology in-country. We confirmed that CHIKV was the dominant pathogen responsible for the outbreak, that DENV was also circulating with 1 in 5 CHIKV positive patients co-infected and, importantly, that there was a high frequency of *falciparum* malaria transmission and of CHIKV/malaria co-infection. The 14 cases with chikungunya RT-PCR negative and ELISA IgM positive results were not unexpected given that the diagnostic window for IgM and RT-PCR are different (IgM antibodies emerge later during the infection and last for longer [34] than the viraemic phase when RNA is identified by RT-PCR). Other possible explanations are prior CHIKV infection but unrelated to this outbreak (as IgM may persist for months [34]) or a false assay result. As IgM/IgG results cannot confirm a diagnosis they were not used to define Chikungunya infection in any analyses.

The cohort recruited were young (median age 27) and largely presented with non-specific febrile symptoms plus poly-articular joint pain. Nevertheless, based on our follow-up cohort, a substantial proportion—one in four—were admitted to hospital, reflecting the impact of the high frequency of malaria co-infection (60%) as well as the severe phenotype observed in a subset of patients, including the potential for haemodynamic disturbance in adults. As we have described, 5% of patients with CHIKV (*P*. *falciparum* and DENV negative) had evidence of bleeding and loss of haemostasis. Two children in particular were gravely unwell with life-threatening haemorrhage associated with severe thrombocytopenia and need for blood product resuscitation, while one adult died of multi-organ failure including acute kidney injury.

Severe and fatal CHIKV infection has been reported previously during outbreaks challenging the misconception that it is only a mild self-limiting diseases.[13,41] Increased risk of severe disease has been suggested in extremes of age (neonates and > 65 years) and those with underlying medical problems including immunosuppression.[10,15] Severe disease has been associated with neurological complications such as acute flaccid paralysis and Guillain-Barré syndrome.[42–44] Brazil, Reunion Island and India all reported higher case fatality and excess deaths during their epidemics, [12,13,16,21,22] though fatality is still considered rare, occurring in less than 1 in 1000 cases.

Although we did not find associations between severe illness and age in our study, or with chronic illness, due to our age-limited follow-up, the findings described by previous studies are consistent with the severe phenotype of CHIKV we observed in Kassala, including a one confirmed CHIKV RT-PCR-positive death and life-threatening bleeding due to loss of haemostasis with thrombocytopenia. The high frequency of *P*. *falciparum* infection detected underpins the importance of both malaria diagnostics and public messaging in malaria-endemic settings when chikungunya outbreaks occur.

Our study was biased towards more severe cases due to hospital-based recruitment and had a low response rate in follow-up where we also lacked a control group. There was a low reported sensitivity of 76.7% (23/30) for the CHIKV IgG ELISA in the convalescent samples tested but it was not possible to determine whether this was due to assay failures or other factors, in part, due to the inability to retest original acute samples to verify results.

However, our findings that 63% of respondents had persistent pain three to four months after acute illness are similar to those of a case-controlled study on La Reunion Island. This study found 53% of IOL-strain CHIKV-positive participants reported twice as much pain compared to controls 12 months after their illness.[45] Our findings were also similar to those of a meta-analysis of chronic disability across all CHIKV strains which found on average 52% of IOL-strain patients were still disturbed by symptoms at three months, compared to 39% of Asian lineage and 14% of ECSA group patients.[24]

Our finding that the only significant risk factor for contracting CHIKV was proximity to a case suggests identifying and supporting vector control measures acceptable to the community should be a priority for public health authorities seeking to prevent future CHIKV and other *Aedes*-transmitted epidemics in Sudan. Entomological research carried out by Kassala University in 2014/15 showed high *Ae*. *aegypti* density across the city and a high proportion of households storing water in their compounds in un-protected clay pots.[46–48] In 2018 the situation was exacerbated by intense seasonal rainfall which displaced 50,000 residents and flooded many areas, a situation repeated in 2020.

Measures such as improved water infrastructure, covered water storage containers, and increased population awareness of the importance of preventing mosquitoes from breeding in household compounds are important.[46] Heightened vector surveillance is also needed to monitor for presence of *Ae*. *albopictus*, which could prompt further outbreaks of different CHIKV strains, and to investigate the potential role in the sylvatic cycle of the large population of Macaques monkeys which live close to residential areas.

Finally, but importantly, it is essential that State Ministries of Health throughout Sudan enhance their risk communication and public information strategies around Chikungunya to underline that severe and occasionally fatal infection exists. With widespread presence of *Ae*. *aegypti* and a similar water storage practices throughout Sudan, timely official and community actions will be needed to prevent another large outbreak in the near future.

ENDS

## Supporting information

S1 STROBE checklist (DOC)

S1 Text Phylogenetic tree showing viral adaptation to different vector species. (DOCX)

## References

[1] Bonney JH, Osei-Kwasi M, Adiku TK, Barnor JS, Amesiya R, Kubio C, et al. Hospital-based surveillance for viral hemorrhagic fevers and hepatitides in Ghana. PLoS neglected tropical diseases. 2013;7(9):e2435. 10.1371/journal.pntd.0002435 24069490 PMC3777898

[2] Russo G, Subissi L, Rezza G. Chikungunya fever in Africa: a systematic review. Pathogens and Global Health. 2020;114(3):111–9. 10.1080/20477724.2020.1743087 32308158 PMC7241529

[3] Humphrey JM, Cleton NB, Reusken CBEM, Glesby MJ, Koopmans MPG, Abu-Raddad LJ. Urban Chikungunya in the Middle East and North Africa: A systematic review. PLoS neglected tropical diseases. 2017;11(6):e0005707. 10.1371/journal.pntd.0005707 28651007 PMC5501693

[4] Robinson MC. An epidemic of virus disease in Southern Province, Tanganyika territory, in 1952–1953. Transactions of the Royal Society of Tropical Medicine and Hygiene. 1955;49(1):28–32. 10.1016/0035-9203(55)90080-8 14373834

[5] Ng LC, Hapuarachchi HC. Tracing the path of Chikungunya virus—evolution and adaptation. Infect Genet Evol. 2010;10(7):876–85. 10.1016/j.meegid.2010.07.012 20654736

[6] Rossi G, Karki S, Smith RL, Brown WM, Ruiz MOH. The spread of mosquito-borne viruses in modern times: A spatio-temporal analysis of dengue and chikungunya. Spatial and Spatio-temporal Epidemiology. 2018;26:113–25. 10.1016/j.sste.2018.06.002 30390927

[7] Leroy EM, Nkoghe D, Ollomo B, Nze-Nkogue C, Becquart P, Grard G, et al. Concurrent chikungunya and dengue virus infections during simultaneous outbreaks, Gabon, 2007. Emerging infectious diseases. 2009;15(4):591–3. 10.3201/eid1504.080664 19331740 PMC2671412

[8] Staples JE, Breiman RF, Powers AM. Chikungunya fever: an epidemiological review of a re-emerging infectious disease. Clinical infectious diseases: an official publication of the Infectious Diseases Society of America. 2009;49(6):942–8. 10.1086/605496 19663604

[9] Pezzi L, Diallo M, Rosa-Freitas MG, Vega-Rua A, Ng LFP, Boyer S, et al. GloPID-R report on chikungunya, o’nyong-nyong and Mayaro virus, part 5: Entomological aspects. Antiviral research. 2020;174:104670. 10.1016/j.antiviral.2019.104670 31812638

[10] van Aalst M, Nelen CM, Goorhuis A, Stijnis C, Grobusch MP. Long-term sequelae of chikungunya virus disease: A systematic review. Travel medicine and infectious disease. 2017;15:8–22. 10.1016/j.tmaid.2017.01.004 28163198

[11] Paixão ES, Teixeira MG, Rodrigues LC. Zika, chikungunya and dengue: the causes and threats of new and re-emerging arboviral diseases. BMJ Global Health. 2018;3(Suppl 1):e000530. 10.1136/bmjgh-2017-000530 29435366 PMC5759716

[12] Burt FJ, Chen W, Miner JJ, Lenschow DJ, Merits A, Schnettler E, et al. Chikungunya virus: an update on the biology and pathogenesis of this emerging pathogen. The Lancet Infectious Diseases. 2017;17(4):e107–e17. 10.1016/S1473-3099(16)30385-1 28159534

[13] Rajapakse S, Rodrigo C, Rajapakse A. Atypical manifestations of chikungunya infection. Transactions of the Royal Society of Tropical Medicine and Hygiene. 2010;104(2):89–96. 10.1016/j.trstmh.2009.07.031 19716149

[14] Mehta R, Gerardin P, de Brito CAA, Soares CN, Ferreira MLB, Solomon T. The neurological complications of chikungunya virus: A systematic review. Reviews in medical virology. 2018;28(3):e1978–e. 10.1002/rmv.1978 29671914 PMC5969245

[15] Economopoulou A, Dominguez M, Helynck B, Sissoko D, Wichmann O, Quenel P, et al. Atypical Chikungunya virus infections: clinical manifestations, mortality and risk factors for severe disease during the 2005–2006 outbreak on Réunion. Epidemiology and Infection. 2009;137(4):534–41. 10.1017/S0950268808001167 18694529

[16] Lemant J, Boisson V, Winer A, Thibault L, Andre H, Tixier F, et al. Serious acute chikungunya virus infection requiring intensive care during the Reunion Island outbreak in 2005–2006. Crit Care Med. 2008;36(9):2536–41. 10.1097/CCM.0b013e318183f2d2 18679124

[17] Contopoulos-Ioannidis D, Newman-Lindsay S, Chow C, LaBeaud AD. Mother-to-child transmission of Chikungunya virus: A systematic review and meta-analysis. PLoS neglected tropical diseases. 2018;12(6):e0006510. 10.1371/journal.pntd.0006510 29897898 PMC6075784

[18] Gérardin P, Sampériz S, Ramful D, Boumahni B, Bintner M, Alessandri J-L, et al. Neurocognitive Outcome of Children Exposed to Perinatal Mother-to-Child Chikungunya Virus Infection: The CHIMERE Cohort Study on Reunion Island. PLoS neglected tropical diseases. 2014;8(7):e2996. 10.1371/journal.pntd.0002996 25033077 PMC4102444

[19] Innovations CfEP. The world needs a chikungunya vaccine [Available from: https://cepi.net/news_cepi/the-world-needs-a-chikungunya-vaccine/.

[20] Lima STSd, Souza WMd, Cavalcante JW, da Silva Candido D, Fumagalli MJ, Carrera J-P, et al. Fatal outcome of chikungunya virus infection in Brazil. Clinical Infectious Diseases. 2020. 10.1093/cid/ciaa1038 32766829 PMC8492446

[21] Mavalankar D, Shastri P, Bandyopadhyay T, Parmar J, Ramani KV. Increased mortality rate associated with chikungunya epidemic, Ahmedabad, India. Emerg Infect Dis. 2008;14(3):412–5. 10.3201/eid1403.070720 18325255 PMC2570824

[22] Josseran L, Paquet C, Zehgnoun A, Caillere N, Le Tertre A, Solet J-L, et al. Chikungunya disease outbreak, Reunion Island. Emerging infectious diseases. 2006;12(12):1994–5. 10.3201/eid1212.060710 17354339 PMC3291364

[23] Freitas ARR, Donalisio MR, Alarcón-Elbal PM. Excess Mortality and Causes Associated with Chikungunya, Puerto Rico, 2014–2015. Emerging infectious diseases. 2018;24(12):2352–5. 10.3201/eid2412.170639 30277456 PMC6256393

[24] Paixao ES, Rodrigues LC, Costa M, Itaparica M, Barreto F, Gerardin P, et al. Chikungunya chronic disease: a systematic review and meta-analysis. Transactions of the Royal Society of Tropical Medicine and Hygiene. 2018;112(7):301–16. 10.1093/trstmh/try063 30007303

[25] Bower H, El Karsany M, Alzain M, Gannon B, Mohamed R, Mahmoud I, et al. Detection of Crimean-Congo Haemorrhagic Fever cases in a severe undifferentiated febrile illness outbreak in the Federal Republic of Sudan: A retrospective epidemiological and diagnostic cohort study. PLoS neglected tropical diseases. 2019;13(7):e0007571. 10.1371/journal.pntd.0007571 31291242 PMC6645580

[26] Markoff L. Yellow fever outbreak in Sudan. The New England journal of medicine. 2013;368:689–91. 10.1056/NEJMp1300772 23387798

[27] World Health Organisation. Dengue Fever—Republic of the Sudan 2019 [Available from: https://www.who.int/csr/don/22-november-2019-dengue-sudan/en/.

[28] American College of Rheumatology. Routine Assessment of Patient Index Data 3 (RAPID3) [Available from: https://www.rheumatology.org/Portals/0/Files/RAPID3%20Form.pdf.

[29] International Severe Acute Respiratory and Emerging Infections Consortium, World Health Organisation. ISARIC-WHO Clinical Characterization Protocol for Severe Emerging Infections (v.3 2014) [Available from: https://isaric.tghn.org/protocols/clinical-characterization-protocol/.

[30] Kafetzopoulou LE, Efthymiadis K, Lewandowski K, Crook A, Carter D, Osborne J, et al. Assessment of metagenomic Nanopore and Illumina sequencing for recovering whole genome sequences of chikungunya and dengue viruses directly from clinical samples. Euro surveillance: bulletin Europeen sur les maladies transmissibles = European communicable disease bulletin. 2018;23(50):1800228. 10.2807/1560-7917.ES.2018.23.50.1800228 30563591 PMC6299504

[31] Ondov BD, Starrett GJ, Sappington A, Kostic A, Koren S, Buck CB, et al. Mash Screen: high-throughput sequence containment estimation for genome discovery. Genome biology. 2019;20(1):232. 10.1186/s13059-019-1841-x 31690338 PMC6833257

[32] Katoh K, Standley DM. MAFFT Multiple Sequence Alignment Software Version 7: Improvements in Performance and Usability. Molecular Biology and Evolution. 2013;30(4):772–80. 10.1093/molbev/mst010 23329690 PMC3603318

[33] Stamatakis A. RAxML version 8: a tool for phylogenetic analysis and post-analysis of large phylogenies. Bioinformatics. 2014;30(9):1312–3. 10.1093/bioinformatics/btu033 24451623 PMC3998144

[34] Chelluboina S, Robin S, Aswathyraj S, Arunkumar G. Persistence of antibody response in chikungunya. Virusdisease. 2019;30(3):469–73. 10.1007/s13337-019-00534-5 31803816 PMC6863990

[35] Tsetsarkin KA, Vanlandingham DL, McGee CE, Higgs S. A Single Mutation in Chikungunya Virus Affects Vector Specificity and Epidemic Potential. PLOS Pathogens. 2007;3(12):e201. 10.1371/journal.ppat.0030201 18069894 PMC2134949

[36] Lindh E, Argentini C, Remoli ME, Fortuna C, Faggioni G, Benedetti E, et al. The Italian 2017 Outbreak Chikungunya Virus Belongs to an Emerging Aedes albopictus-Adapted Virus Cluster Introduced From the Indian Subcontinent. Open forum infectious diseases. 2018;6(1):ofy321–ofy. 10.1093/ofid/ofy321 PMC6345083 30697571

[37] Sergon K, Yahaya AA, Brown J, Bedja SA, Mlindasse M, Agata N, et al. Seroprevalence of Chikungunya virus infection on Grande Comore Island, union of the Comoros, 2005. Am J Trop Med Hyg. 2007;76(6):1189–93. 17556634

[38] Sergon K, Njuguna C, Kalani R, Ofula V, Onyango C, Konongoi LS, et al. Seroprevalence of Chikungunya virus (CHIKV) infection on Lamu Island, Kenya, October 2004. Am J Trop Med Hyg. 2008;78(2):333–7. 18256441

[39] Sissoko D, Malvy D, Giry C, Delmas G, Paquet C, Gabrie P, et al. Outbreak of Chikungunya fever in Mayotte, Comoros archipelago, 2005–2006. Transactions of the Royal Society of Tropical Medicine and Hygiene. 2008;102(8):780–6. 10.1016/j.trstmh.2008.02.018 18400240

[40] Renault P, Solet JL, Sissoko D, Balleydier E, Larrieu S, Filleul L, et al. A major epidemic of chikungunya virus infection on Reunion Island, France, 2005–2006. Am J Trop Med Hyg. 2007;77(4):727–31. 17978079

[41] Suhrbier A. Rheumatic manifestations of chikungunya: emerging concepts and interventions. Nature Reviews Rheumatology. 2019;15(10):597–611. 10.1038/s41584-019-0276-9 31481759

[42] Sá PKdO, Nunes MM, Leite I, Campelo M, Leão C, Souza JdC, et al. Chikungunya virus infection with severe neurologic manifestations: report of four fatal cases. Revista da Sociedade Brasileira de Medicina Tropical. 2017;50:265–8. 10.1590/0037-8682-0375-2016 28562768

[43] Wielanek AC, Monredon JD, Amrani ME, Roger JC, Serveaux JP. Guillain-barré syndrome complicating a chikungunya virus infection. Neurology. 2007;69(22):2105. 10.1212/01.wnl.0000277267.07220.88 18040016

[44] Singh SS, Manimunda SP, Sugunan AP, Sahina, Vijayachari P. Four cases of acute flaccid paralysis associated with chikungunya virus infection. Epidemiology and infection. 2008;136(9):1277–80. 10.1017/S0950268807009739 18634716 PMC2870928

[45] Soumahoro M-K, Gérardin P, Boëlle P-Y, Perrau J, Fianu A, Pouchot J, et al. Impact of Chikungunya Virus Infection on Health Status and Quality of Life: A Retrospective Cohort Study. PLOS ONE. 2009;4(11):e7800. 10.1371/journal.pone.0007800 19911058 PMC2771894

[46] Ahmed R, Hassan S, Abdallah K, Enan M. Breeding and Resting Behaviour of Aedes aegypti in Indoor and Outdoor Environment in Kassala City, Sudan 2014/2015. Health Science Journal. 2019;13 (5: 672).

[47] Ahmed R, Hassan S. Seasonal indices of Aedes aegypti (Diptera: Culicidae) in an urban area of Kassala City, Sudan, 2014–2015. Europ Acad Res. 2019;V1(10).

[48] Ahmed R, Hassan S, Elrahman A. Climatic Factors Affecting Density of Aedes aegypti (Diptera: Culicidae) in Kassala City, Sudan 2014/2015. Asploro Journal of Biomedical and Clinical Case Reports. 2019;2(2):58–68.

