# Supplementary material for: “Kankasha” in Kassala: A prospective observational cohort study of the clinical characteristics, epidemiology, genetic origin, and chronic impact of the 2018 epidemic of Chikungunya virus infection in Kassala, Sudan
Source: PLoS Negl Trop Dis. 2021 Apr 30;15(4):e0009387. doi: 10.1371/journal.pntd.0009387 (PMC8115788; doi:10.1371/journal.pntd.0009387)
Supplement: S1 STROBE checklist — (DOC) [file pntd.0009387.s001.doc]

STROBE Statement—Checklist of items that should be included in reports of ***cohort studies***

|  | | Item No | Recommendation |
| --- | --- | --- | --- |
| **Title and abstract** | | 1 | (*a*) Indicate the study’s design with a commonly used term in the title or the abstract  **L2**: a prospective observational cohort study of the clinical characteristics, epidemiology, genetic origin, and chronic impact of… |
|  |  |  | (*b*) Provide in the abstract an informative and balanced summary of what was done and what was found  **L60-86** |
| Introduction | | | |
| Background/rationale | | 2 | Explain the scientific background and rationale for the investigation being reported  **L107-144** |
| Objectives | | 3 | State specific objectives, including any prespecified hypotheses  **L150:** to investigate the outbreak syndrome, confirm the outbreak pathogen, and sequence the outbreak strain. |
| Methods | | | |
| Study design | | 4 | Present key elements of study design early in the paper  **L171:** The study was a prospective hospital-based observational cohort of consecutive patients presenting with UFI (case definition, Figure 2) The sample size of 140 cases was informed by existing literature and feasible recruitment in the urgent time frame (7 days). |
| Setting | | 5 | Describe the setting, locations, and relevant dates, including periods of recruitment, exposure, follow-up, and data collection  **L 172:** at the KTH medical and paediatrics outpatient clinics departments between 10^th^ and 16^th^ October 2018. Epidemiological information, clinical symptoms and laboratory results were recorded at presentation on standardised pro-formas.  **L177**: Blood samples were drawn at initial assessment, and adults provided convalescent samples and completed the WHO-validated Routine Assessment of Patient Index Data 3 (RAPID3) disability and pain survey 90-120 days later (Jan-Feb 2019). |
| Participants | | 6 | (*a*) Give the eligibility criteria, and the sources and methods of selection of participants. Describe methods of follow-up  **L171:** cohort of consecutive patients presenting with UFI (case definition, Figure 2) at the KTH medical and paediatrics outpatient clinics departments between 10^th^ and 16^th^ October 2018  **L176:** adults provided convalescent samples and completed the WHO-validated Routine Assessment of Patient Index Data 3 (RAPID3) disability and pain survey^26^ 90-120 days later (Jan-Feb 2019). Children were not asked to return due to common reluctance to allow blood-draw from healthy children, but outcome and duration of hospital stay were confirmed with parents. |
|  |  |  | (*b*) For matched studies, give matching criteria and number of exposed and unexposed  ***N/A*** |
| Variables | | 7 | Clearly define all outcomes, exposures, predictors, potential confounders, and effect modifiers. Give diagnostic criteria, if applicable  **L183-193 & 298 :** Biochemistry, diagnostic assay and clinical parameters  **L235**: Table 1 Demographic variables  **L264**: Table 2 Clinical variables  **L353**: Table 4 Disability and chronic pain variables |
| Data sources/ measurement | | 8* | For each variable of interest, give sources of data and details of methods of assessment (measurement). Describe comparability of assessment methods if there is more than one group  **Exposed and unexposed here relates to chikungunya infection and was not known before recruitment*  ***L183****:* Haematology (Mindaray 3000 Plus, China), biochemistry analysis (Biosystem BTS 310, Germany), and malaria rapid diagnostic tests (RDT, SD Bioline, USA) were performed at admission on all participants: malaria thick film examination was done for all children.  **L 173**: Epidemiological information, clinical symptoms and laboratory results were recorded at presentation on standardised pro-formas  **L177**: WHO-validated Routine Assessment of Patient Index Data 3 (RAPID3) disability and pain survey^26^ 90-120 days later (Jan-Feb 2019). |
| Bias | | 9 | Describe any efforts to address potential sources of bias  **L427**: Our study was biased towards more severe cases due to hospital-based recruitment and a low response rate among and lack of a control for participants followed up within 90-120 days.  **L429** : **we compare our findings with those from a controlled study in a large outbreak and a meta-analysis of chronic disability. It was not possible during the outbreak and in the time available to address bias in the study itself* |
| Study size | | 10 | Explain how the study size was arrived at  **L175**: The sample size of 140 cases was informed by existing literature3 and feasible recruitment in the urgent time frame (7 days). |
| Quantitative variables | | 11 | Explain how quantitative variables were handled in the analyses. If applicable, describe which groupings were chosen and why  **L196**: expressed as medians and Inter-Quartile Range (IQR) or means and standard deviations  **L236**: *Age is grouped initially in 2-year period (<2 & 2-4) to capture younger age in more detail, then by 10 years until 50 years, with 50+ as one group to prevent small numbers* |
| Statistical methods | | 12 | (*a*) Describe all statistical methods, including those used to control for confounding  **L196**: Descriptive statistics are expressed as medians and Inter-Quartile Range (IQR) or means and standard deviations (SD) for continuous variables, and frequencies and proportions for categorical variables. Fisher’s exact, Chi^2^, T-tests and Spearman’s Rank Order coefficients were used to assess association and correlation. Significance was set at p<0.05. |
|  |  |  | (*b*) Describe any methods used to examine subgroups and interactions  **L198**: Fisher’s exact, Chi^2^, T-tests and Spearman’s Rank Order coefficients were used to assess association and correlation related to CHIKV PCR result. Significance was set at p<0.05. |
|  |  |  | (*c*) Explain how missing data were addressed  *Denominators and missing data specified per variable throughout* |
|  |  |  | (*d*) If applicable, explain how loss to follow-up was addressed  **L177**: adults who responded to phone follow-up provided convalescent samples and completed the WHO-validated Routine Assessment of Patient Index Data 3 (RAPID3) disability and pain survey 90-120 days later (Jan-Feb 2019). Children were not asked to return due to common reluctance to allow blood-draw from healthy children, |
|  |  |  | (*e*) Describe any sensitivity analyses  **N/A** |
| Results | | | |
| Participants | 13* | (a) Report numbers of individuals at each stage of study—eg numbers potentially eligible, examined for eligibility, confirmed eligible, included in the study, completing follow-up, and analysed  **L227**: Figure 3: Flow diagram of study participants and virological finding**s** | |
|  |  | (b) Give reasons for non-participation at each stage  **As above and L 224**: A total of 102 adults and 42 children (<18 years) were recruited over seven days, of which two cases without samples were excluded. 11 patients or their guardians refused to participate, and 2 patients dead on arrival were not included as sampling was not possible (Figure 3). | |
|  |  | (c) Consider use of a flow diagram  **As above** | |
| Descriptive data | 14* | (a) Give characteristics of study participants (eg demographic, clinical, social) and information on exposures and potential confounders  **L229**: Participants were aged 4 months to 70 years (mean 27 years, SD 17.6); 47.9% were female, none were pregnant (Table 1). Almost half (46.1%, 65/141) were people likely to spend more time in a household compound (e.g. housewives, unemployed, retired, children under 5). Median household size was 8. Two-thirds of participants lived in brick/concrete houses with own well and sanitation, the remainder lived in less permanent material structures with shared water and sanitation. A third kept animals in their compound.  **L239**: Most participants came from Kassala City sectors 2,3, 4 and 5 on the banks of the seasonal River Gash (69%, 80/116), coinciding with areas of greatest flooding in the 2018 rainy season, and with highest case reports during the epidemic (Figure 4). A further 26% (30/116) were from rural areas up to 1.5 hours’ drive away.  **L251**: Median delay from symptom onset to presentation was 2 days (IQR 1-4, n=139) with no difference by CHIKV diagnosis or age (Table 2). Sixteen (26.6%) participants were admitted (4 adults, 12 children). Children were more likely to be admitted than adults and for longer: a mean of 4.7 days compared to 2.3 days for adults (p=0.04).  Most common symptoms at presentation among CHIKV PCR-positive participants were fever (97.5%), headache (88.2%), fatigue (82.5%), muscle, joint and back pain (66.0%, 83.8%, 50.9% respectively), loss of appetite (40.4%) and vomiting (40.2%); patients who were CHIKV PCR-negative had similar presenting symptoms. Participants co-infected with CHIKV and DENV were more likely to present with back pain than those with CHIKV alone (p=0.003). Five percent (6/120) of CHIKV PCR-positive participants reported bleeding, including haematemesis (4), oral bleeding (2), epistaxis (3), petechiae (1), haemoptysis (1) and melaena (1). Ten percent of CHIKV PCR-positive adults (18/78) were hypotensive (systolic blood pressure <100 mmHg) with seven also being tachycardic (pulse >100).  **L183-193 & 298** : Biochemistry, diagnostic assay and clinical parameters  **L235**: Table 1 Demographic variables  **L264**: Table 2 Clinical variables | |
|  |  | (b) Indicate number of participants with missing data for each variable of interest  *Missing data and denominators are indicated throughout* | |
|  |  | (c) Summarise follow-up time (eg, average and total amount)  **L328**: Thirty (29.7%) of the 102 adult participants could be followed-up 90-120 days after enrolment, 29 (28.7%) refused, and 42 (41.6%) could not be reached. Parents of 30 of the 40 child participants responded to contact. | |
| Outcome data | 15* | Report numbers of outcome events or summary measures over time  **L269**: Of 142 participants, 120 were confirmed positive for CHIKV infection by qRT-PCR,  **L277**: Nineteen percent of CHIKV PCR-positive participants (23/120) were coinfected with DENV either by PCR or IgM-ELISA. Of all samples (CHIKV-positive and negative), 81.6% (115/141) were DENV IgG-positive indicating previous infection. Of the 118 CHIKV PCR-positive participants with malaria RDT result, 28.8% were positive, with the highest proportion in those aged 15-29 years. No participants were co-infected with all three pathogens.  **L359**: Virus genetic sequencing found that all CHIKV PCR-positive samples belonged to a single monophyletic cluster in the Indian Ocean Lineage (IOL) of the ECSA genotype of CHIKV (Figure 4) | |
| Main results | 16 | (*a*) Give unadjusted estimates and, if applicable, confounder-adjusted estimates and their precision (eg, 95% confidence interval). Make clear which confounders were adjusted for and why they were included  *No estimates or adjusted analysis is reported* | |
|  |  | (*b*) Report category boundaries when continuous variables were categorized  **L291**: Leucopenia (white blood count < 4 x 10^9^/L) was observed in 29% (35/120) and lymphopenia (< 1 x 10^9^/L) observed in 50.7% (61/120). Elevated AST (>40IU/L) was observed in 22.5% (27/120) with six participants at levels> 100 IU/L (max 259 IU/L). ALT levels >51 IU/L were seen in seven participants with four recording levels >100 (max 150 IU/l). Acute kidney injury was observed in one fatal case. Platelet counts < 100 x 10^9^/L were recorded in 23/120 CHIKV PCR-positive participants, of whom 14/23 were also malaria RDT positive and none DENV PCR positive. Platelet counts < 50 x 10^9^/L were observed in six participants, three of whom also had positive malaria RDT. | |
|  |  | (*c*) If relevant, consider translating estimates of relative risk into absolute risk for a meaningful time period  **N/A** | |
| Other analyses | 17 | Report other analyses done—eg analyses of subgroups and interactions, and sensitivity analyses  **L282**: Among participants with matched CHIKV PCR and ELISA results, 31/69 PCR-positive were also IgM-positive, and 34/119 (28.6%) were CHIKV IgG positive (Figure 3). Participants who were CHIKV positive on both PCR and IgM presented later in illness course than those who were CHIKV PCR-positive and IgM-negative (p<0.001). This finding was replicated with CHIKV PCR & IgG-positive patients who presented later than CHIKV IgG-negative patients (p=0.002).  **L321**: The mean age of CHIKV PCR-positive patients was significantly less than CHIKV PCR-negative patients (p=0.003). CHIKV PCR-positives were also more likely to report exposure to someone who was ill (p=0.01) … No associations were found with any other exposure. Compared to those with a single infection, CHIKV/DENV coinfected participants had higher mean systolic blood pressure (p=0.03) haemoglobin (p=0.01), haematocrit (p=0.03), bilirubin (p=0.002) and albumin (p=<0.001). | |
| Discussion | | | |
| Key results | 18 | Summarise key results with reference to study objectives  **L391**: Our phylogenetic analysis suggests the unprecedented Chikungunya epidemic that took place in the Eastern states of Sudan in 2018-19 was caused by an independent introduction of a CHIKV IOL virus, of the same strain responsible for the outbreaks observed worldwide during 2004-2007 and linked to more severe manifestations of CHIKV disease.  **L408**: The cohort recruited were young (median age 27) and largely presented with non-specific febrile symptoms plus poly-articular joint pain. The substantial proportion (26%) admitted to hospital, however, reflects high frequencies of co-infection and the potential for haemodynamic disturbance in adults, and other manifestations of a severe phenotype observed in a subset of patients. As we have described, 5% of patients with CHIKV (malaria and DENV negative) had evidence of bleeding and loss of haemostasis. Two children in particular were gravely unwell with life-threatening haemorrhage associated with severe thrombocytopenia and need for blood product resuscitation, while one adult died of multi-organ failure including acute kidney injury.  **L423**: These findings are consistent with the severe phenotype of CHIKV we observed in Kassala, including a one confirmed CHIKV PCR-positive death and life-threatening bleeding due to loss of haemostasis with thrombocytopenia. Additionally, the high frequency of *P.falciparum* infection detected underpins the importance of both malaria diagnostics and public messaging in malaria-endemic settings when chikungunya outbreaks occur. | |
| Limitations | 19 | Discuss limitations of the study, taking into account sources of potential bias or imprecision. Discuss both direction and magnitude of any potential bias  **L428**: Our study was biased towards more severe cases due to hospital-based recruitment and a low response rate among and lack of a control for participants followed up within 90-120 days. However, our findings that 63% of respondents had persistent pain three to four months after acute illness are similar to those of a case-controlled study on La Reunion Island. This study found 53% of IOL-strain CHIKV-positive participants reported twice as much pain compared to controls 12 months after their illness.^41^ Our findings were also similar to those of a meta-analysis of chronic disability across all CHIKV strains which found on average 52% of IOL-strain patients were still disturbed by symptoms at three months, compared to 39% of Asian lineage and 14% of ECSA group patients. | |
| Interpretation | 20 | Give a cautious overall interpretation of results considering objectives, limitations, multiplicity of analyses, results from similar studies, and other relevant evidence  **L436**: Our finding that the only significant risk factor for contracting CHIKV was proximity to a case suggests identifying and supporting vector control measures acceptable to the community should be a priority for public health authorities seeking to prevent future CHIKV and other *Aedes*-transmitted epidemics in Sudan  **L449**: it is essential that State Ministries of Health throughout Sudan enhance their risk communication and public information strategies around Chikungunya to underline that severe and occasionally fatal infection exists. The epidemic we describe caused substantial health and economic burden for the affected populations. With widespread presence of *Ae. aegypti* and a similar water storage practices throughout Sudan, timely actions will be needed to prevent another large outbreak in the near future. | |
| Generalisability | 21 | Discuss the generalisability (external validity) of the study results  **L452**: The epidemic we describe caused substantial health and economic burden for the affected populations. With widespread presence of *Ae. aegypti* and a similar water storage practices throughout Sudan, timely actions will be needed to prevent another large outbreak in the near future. | |
| Other information | | | |
| Funding | 22 | Give the source of funding and the role of the funders for the present study and, if applicable, for the original study on which the present article is based  **L46**: The study was funded by UK aid from the Department of Health and Social Care (<https://www.gov.uk/government/collections/official-development-assistance-oda--2>, Grant No. IS-RRT-1015-001) via the UK Public Health Rapid Support Team Research Programme (Grant No. RST3_03). The funder had no role in study design, data collection and analysis, decision to publish or preparation of the manuscript. | |

*Give information separately for exposed and unexposed groups.

**Note:** An Explanation and Elaboration article discusses each checklist item and gives methodological background and published examples of transparent reporting. The STROBE checklist is best used in conjunction with this article (freely available on the Web sites of PLoS Medicine at http://www.plosmedicine.org/, Annals of Internal Medicine at http://www.annals.org/, and Epidemiology at http://www.epidem.com/). Information on the STROBE Initiative is available at http://www.strobe-statement.org.
