# Supplementary material for: “Kankasha” in Kassala: A prospective observational cohort study of the clinical characteristics, epidemiology, genetic origin, and chronic impact of the 2018 epidemic of Chikungunya virus infection in Kassala, Sudan
Source: PLoS Negl Trop Dis. 2021 Apr 30;15(4):e0009387. doi: 10.1371/journal.pntd.0009387 (PMC8115788; doi:10.1371/journal.pntd.0009387)
Supplement: S1 Text — (DOCX) [file pntd.0009387.s002.docx]

**CHIKV ECSA phylogenetic tree showing amino acid identities associated with viral adaptation to different vector species.**


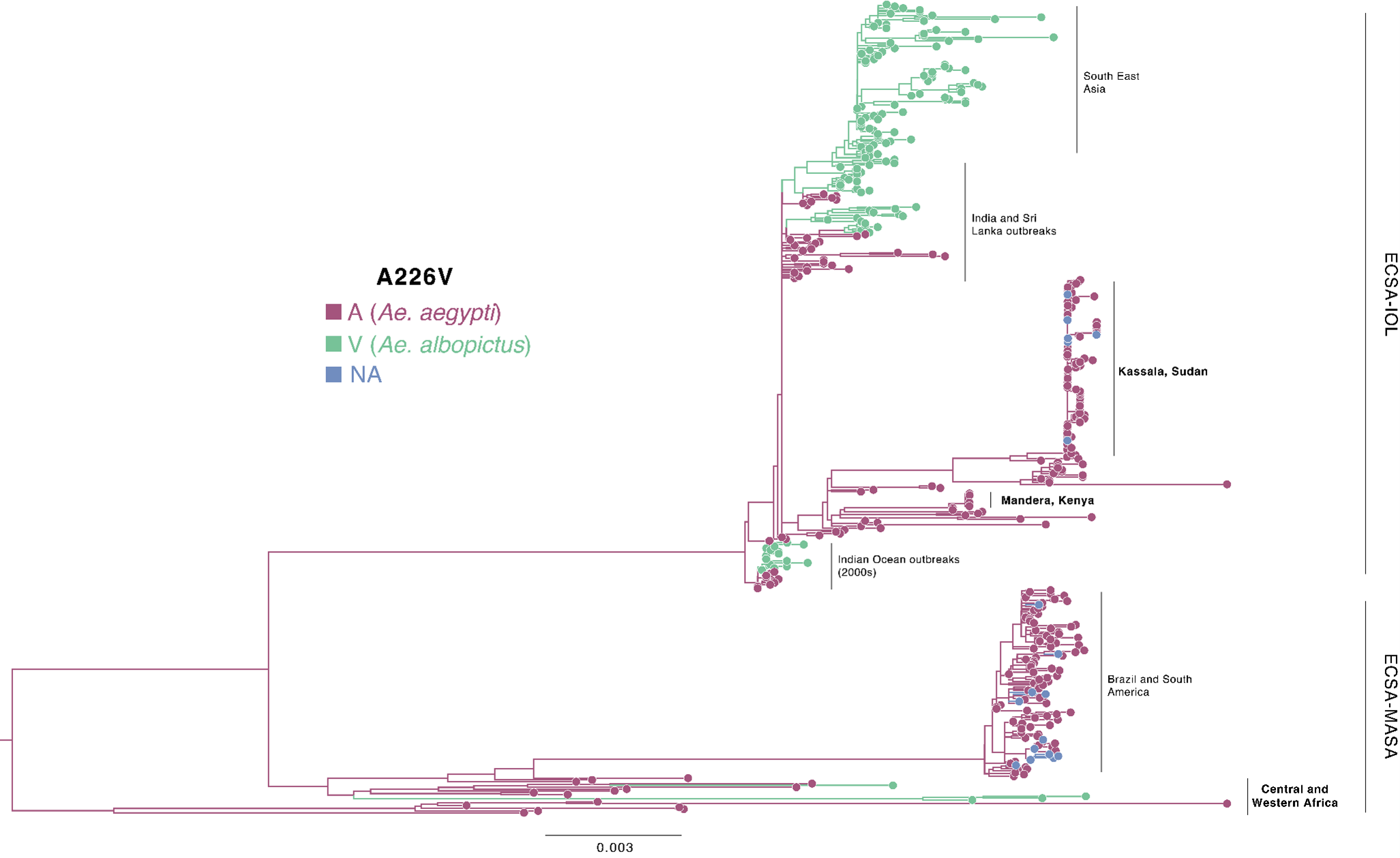


**Notes:**  *The amino acid identity at site 226 of the E1 protein is associated with viral adaptation to different vector species. Viruses containing the A226 variant infect Ae. aegypti more efficiently, while viruses containing the V226 variant more efficiently infect Ae. albopictus. The evolution of this trait is mapped (through a parsimony reconstruction) on the tree in different colours.*
